# Knowledge on HPV Vaccine and Cervical Cancer Facilitates Vaccine Acceptability among School Teachers in Kitui County, Kenya

**DOI:** 10.1371/journal.pone.0135563

**Published:** 2015-08-12

**Authors:** Moses Muia Masika, Javier Gordon Ogembo, Sophie Vusha Chabeda, Richard G. Wamai, Nelly Mugo

**Affiliations:** 1 Department of Medical Microbiology, School of Medicine, University of Nairobi, Nairobi, Kenya; 2 Department of Medicine, University of Massachusetts Medical School, Worcester, Massachusetts, United States of America; 3 Partners in Health Research and Development, Thika, Kenya; 4 Department of African-American Studies, Northeastern University, Boston, Massachusetts, United States of America; 5 Kenya Medical Research Institute (KEMRI), Nairobi, Kenya; Universidad Nacional de La Plata., ARGENTINA

## Abstract

**Background:**

Vaccines against human papillomavirus (HPV) infection have the potential to reduce the burden of cervical cancer. School-based delivery of HPV vaccines is cost-effective and successful uptake depends on school teachers’ knowledge and acceptability of the vaccine. The aim of this study is to assess primary school teachers’ knowledge and acceptability of HPV vaccine and to explore facilitators and barriers of an ongoing Gavi Alliance-supported vaccination program in Kitui County, Kenya.

**Methods:**

This was a cross-sectional, mixed methods study in Central Division of Kitui County where the Ministry of Health is offering the quadrivalent HPV vaccine to grade four girls. Data on primary school teachers’ awareness, knowledge and acceptability of HPV vaccine as well as facilitators and barriers to the project was collected through self-administered questionnaires and two focus group discussions.

**Results:**

339 teachers (60% female) completed the survey (62% response rate) and 13 participated in 2 focus group discussions. Vaccine awareness among teachers was high (90%), the level of knowledge about HPV and cervical cancer among teachers was moderate (48%, SD = 10.9) and females scored higher than males (50% vs. 46%, *p = 0*.*002*). Most teachers (89%) would recommend the vaccine to their daughter or close relatives. Those who would recommend the vaccine had more knowledge than those who would not (*p = <0*.*001*). The main barriers were insufficient information about the vaccine, poor accessibility of schools, absenteeism of girls on vaccine days, and fear of side effects.

**Conclusions:**

Despite low to moderate levels of knowledge about HPV vaccine among school teachers, vaccine acceptability is high. Teachers with little knowledge on HPV vaccine are less likely to accept the vaccine than those who know more; this may affect uptake if not addressed. Empowering teachers to be vaccine champions in their community may be a feasible way of disseminating information about HPV vaccine and cervical cancer.

## Introduction

Human papillomavirus (HPV) is linked to the etiology of various cancers in humans, including oral, pharyngeal, anal and genital cancers [1–4]. Key among these is cervical cancer, which is due to persistent infection with oncogenic HPV genotypes in virtually all cases [5]. Cervical cancer is the third most common cancer in women worldwide [6]. Each year, there are 530,000 cases and 275,000 deaths from cervical cancer globally [7]. Nearly 90% of these are in low-income countries (LICs) [7] and it is the leading cause of cancer deaths in women in sub-Saharan Africa (SSA) [8]. Eastern Africa has the highest burden of cervical cancer in the world, with an age-standardized incidence ratio of 34.5 against an average of 9 per 100,000 in the developed world [8]. In 2012, Kenya had 4,802 cases of cervical cancer, 51% of whom died [9].

Prophylactic vaccinations against HPV infection bear the potential to reduce the burden of cervical cancer and have proven to be cost-effective when offered to women before infection with HPV, especially in LICs where screening strategies are sub-optimal [10–12]. Currently, there are three HPV vaccines that are safe and efficacious in preventing HPV infection: bivalent(Cervarix, GlaxoSmithKline), quadrivalent (Gardasil, Merck), and nonavalent (Gardasil, Merck) HPV vaccines which protects against HPV 16 and 18; HPV 6, 11, 16 and 18; and HPV 6, 11, 16, 18, 31, 33, 45, 52 and 58, respectively [13]. HPV16 and 18 are the primary cause of 70% of all cervical cancers worldwide [14]. HPV 6 and 11 are present in over 90% of all anogenital warts [15].

Studies in SSA show high HPV vaccine acceptability [16–21] but only two countries, Rwanda and recently, South Africa, have been able to roll out a national program [22,23]. To date, at least five SSA countries—Cameroon, Kenya, Uganda, Lesotho, and Tanzania—have piloted delivery of HPV vaccine to adolescent girls using different approaches [21,24–26].

Various avenues have been utilized to deliver HPV vaccine to targeted populations, including schools, health-facilities and community outreach [16,20,22,24,27,28]. The school-based approach has been shown to achieve high vaccine uptake in SSA [16,19,29]. Some countries have also opted to combine the school-based approach with health facility or community outreach in order to broaden vaccine reach, especially for girls who are not enrolled in school [21,22,30,31].

In a school-based approach, teachers play a pivotal role in HPV vaccine delivery. This includes giving permission for use of school premises, educating parents and pupils on the vaccine and organization of vaccine days. Teachers' knowledge and attitude towards the vaccine has been shown to significantly affect the success of school-based HPV vaccination programs [32,33]. Disseminating the correct information about the vaccine is key in ensuring community support. Erosion of public trust due to concerns about vaccine safety and future fertility as well as political and religious factors have slowed down vaccination in some countries like Rwanda and Cameroon [20,21,34,35]. Similar concerns have even led to program suspension in other countries such as Japan [36], India [37] and Canada [38].

For most SSA countries, cost has previously been cited as the biggest impediment to rolling out HPV vaccination on a national scale [39]. In 2012, Gavi Alliance announced a price of USD 4.50 for all Gavi Alliance-eligible countries down from USD120 per dose [40,41]. The Alliance has offered support for national introduction of HPV vaccine for countries with demonstrated ability; or to co-fund demonstration projects for two years. The demonstration projects are to guide planning and implementation of nation-wide HPV vaccination programs that are expected to follow [41,42]. By February 2014, Gavi had approved 21 countries including Kenya for HPV vaccine demonstration program in sub-Saharan Africa [43]. Kenya's Ministry of Health (MOH), with the support of Gavi Alliance, started a two-year HPV vaccination demonstration project in Kitui County for all girls in grade four in both public and private schools. Ten year old girls not enrolled in school were also targeted for vaccination at health facilities and through community outreach [44].

In this study, we assessed the knowledge of HPV vaccine and cervical cancer and the acceptability of HPV vaccination among primary school teachers in Kitui given the use of schools in the campaign. We also explored facilitators and barriers to the uptake and completion of HPV vaccination. Assessing teachers’ knowledge and acceptance of HPV vaccine to identify any gaps that exist is useful to programmers in designing vaccination campaigns. Teachers are also well placed to identify facilitators and barriers of vaccine uptake and opportunities that can be used to mount other health promotion interventions during the vaccination campaign.

## Methodology

### Study design

This is a cross-sectional, mixed-methods approach, using both qualitative and quantitative techniques.

### Study area and population

The study was conducted in Central Division in Kitui County where the MOH has been undertaking HPV vaccination of grade four girls since 2013. Kitui County is one of 47 administrative regions in Kenya. The County’s geographic, social and economic challenges mirror those of a majority of the rest of the country [45,46]. In 2013, 98% of girls in Kitui County were enrolled in school with 8,455 girls in grade four, 94% of whom were 9–13 years old, and 166 ten-year old girls not enrolled in school [44]. The county has about 1,100 primary schools [47]. Due to limited resources, Kitui Central Division was selected to represent the County. The Division has 80 primary schools (73 public and 7 private) and about 700 primary school teachers [47].

### Sampling

Multi-stage sampling was done by stratifying the schools into public and private institutions and then selecting 34 public and 3 private schools through systematic random sampling. All teachers in selected schools were invited to participate in the study by completing a self-administered questionnaire.

### Data collection

We used a self-administered structured questionnaire and focus group discussions to collect data. The questionnaire was designed specifically for this study and did not have identifying data. It was in English, a language the target population understood well. Seventeen questions were used to assess teachers’ knowledge on HPV vaccine and cervical cancer (S1 Text). These were in ‘true/false’ format or multiple choice questions where the respondent was asked to select one or more correct statements. The individual scores were later converted to percentages.

Fourteen of the 37 schools were randomly selected and requested to send the head teacher, deputy head-teacher or HPV vaccination coordinator, to one of two Focus Group Discussions (FGDs). FGDs were held on different days in a hotel meeting room in Kitui Town. They were conducted in English, guided by a semi-structured questionnaire and recorded on a voice recorder.

### Data management

Quantitative data was cleaned and entered into SPSS for analysis. Univariate analysis was done by use of frequency distributions and proportions for categorical variables and descriptive statistics for continuous variables.

Bivariate analysis to test associations was done using Chi-square or Fisher’s Exact Test for categorical variables, and t-test for continuous variables. The level of significance for all tests was set at 5%.

FGDs recordings were transcribed and proofed to eliminate transcription and grammatical errors. The transcriptions were loaded on to ATLAS.ti, coded into thematic groups derived from the FGD questionnaire and actual discussions, and analyzed to identify the strength and pattern of participants’ views.

### Ethics statement

This study was approved by the Kenyatta National Hospital-University of Nairobi Ethics and Research Committee and the National Commission for Science, Technology and Innovation (NACOSTI). Permission to conduct the study in Kitui was granted by the Kitui County Commissioner, Kitui County Director of Education and head-teachers of selected schools. Written informed consent was obtained from all participants.

Data was collected using anonymous questionnaires and no personal identifiers were analyzed or disseminated.

The 13 teachers who participated in the FGDs were reimbursed for transport cost to and from the FGD venue (US$ 12).

## Results

### Respondents’ characteristics

Out of 507 teachers in 37 schools, 339 completed the survey (62% response rate); the rest were unavailable during the time of administering the questionnaire or were unwilling to participate in the study. Sixty percent were female and 85% were working in rural schools. Ninety-nine percent were Christians (Catholics 44% and Protestants 55%). The average age was 40 years (Standard deviation (SD) = 10.7). A majority of the respondents were married (77%) (Table 1).

**Table 1:** Respondents' Demographic Characteristics.

Characteristic		Public Schools	Private Schools	Total (%)
**School Location**	Rural	276	11	**287 (85%)**
	Urban	37	15	**52 (15%)**
	Total	313 (92%)	26 (8%)	**339**
**Sex**	Female	191	11	**202 (60%)**
	Male	117	15	**132 (40%)**
	Total	308 (92%)	26 (8%)	**334**
**Age** (years)	Average	41	28	**40 (SD = 10.7)**
**Age group**	Under 25 years	18	2	**20 (6.5%)**
	25–34 years	62	23	**85 (27.7%)**
	35–44 years	82	0	**82 (26.7%)**
	45–54 years	89	0	**89 (29%)**
	Over 55 years	31	0	**31 (10.1%)**
	Total	282 (92%)	25 (8%)	**307**
**Level of Education**	Secondary	61	0	**61 (18%)**
	Certificate	91	22	**113 (34%)**
	Diploma	86	4	**90 (27%)**
	Degree	69	0	**69 (21%)**
	Total	307 (92%)	26 (8%)	**333**
**Religion**	Protestant	168	18	**185 (55%)**
	Catholic	142	8	**150 (44%)**
	Muslim	3	0	**3 (1%)**
	Total	313 (92%)	26 (8%)	**339**
**Marital Status**	Married	242	17	**259 (77%)**
	Single	61	8	**69 (21%)**
	Other	7	0	**7 (2%)**
	Total	310 (93%)	25 (7%)	**335**

Two FGDs with a total of 13 teachers from 13 primary schools were held. One of the 14 invited schools did not send a representative. Seven of the participants were female, three were head-teachers, three deputy head-teachers and seven were HPV vaccination project coordinators in their respective schools.

### Awareness of the HPV vaccination program

Ninety percent of the teachers were aware that the government had launched the HPV vaccination campaign targeting all grade four girls in Kitui County. Sources of information were MOH officials (68%), fellow teachers (30%), radio (13%), Ministry of Education officials (11%), and television (5%). Teachers in rural schools were more likely to be aware of the initiative than teachers in urban schools (91% vs. 82%, *p =* 0.047). In the FGDs, many suggested that parents and other community members should be informed about the vaccine through *barazas* (public gatherings held by local leaders), churches and other social gatherings, before launching the vaccine initiative.

### Knowledge on HPV vaccine and cervical cancer

The mean score on knowledge on 17 questions asked was 48% (SD = 10.9, range = 12–84%). Women had a higher knowledge score than men *(*50% vs. 46%, *p =* 0.002). There was no significant difference in knowledge score by school type, school location, teachers’ religion, marital status or age.

Ninety-five percent of the participants knew that the HPV vaccine prevents cervical cancer, but they had very little information about HPV infection and cervical cancer. Eighty-four percent understood that cervical cancer is an important disease that kills many women and 61% correctly responded that Pap smear is used for cervical cancer screening. However, they had no knowledge on HPV, its transmission, signs or symptoms. Table 2 shows the proportion of correct responses to each question.

**Table 2:** Questions and the percentage of Correct Responses.

Question	Correct Answer	Correct Responses (%)
HPV Vaccine Protects against HIV	NO	335 (99)
HPV Vaccine Protects against Breast Cancer	NO	333 (98)
HPV Vaccine Protects against Cervical Cancer	YES	323 (95)
Aware of HPV Vaccine exercise in Kitui County	YES	300 (90)
Cervical cancer is a leading cause of cancer deaths in women	YES	284 (84)
HPV causes Cervical Cancer	YES	280 (83)
HPV can be transmitted by aerosol/droplet	NO	271 (80)
Ever heard about Pap smear	YES	238 (70)
Pap Smear is used for cervical cancer screening	YES	261 (61)
HPV can be transmitted through Sexual Contact	YES	197 (58)
There’s no need for Pap Smear after HPV vaccine	NO	156 (46)
Nearly everyone infected with HPV is symptomatic	NO	89 (26)
HPV infects both men and women	YES	26 (8)
HPV can be transmitted through Physical Contact	YES	20 (6)
HPV Vaccine Protects against Vulvar Cancer	YES	18 (5)
HPV Vaccine Protects against Anal Cancer	YES	7 (2)
HPV Vaccine Protects against Warts	YES	3 (1)

Some of the comments from FGD participants are shown below:

*‘I thought it (HPV) may be inborn*, *the child maybe born with it*, *so it maybe still with the child as she grows up and emerge later…’*

*‘I think from the word cervix*, *one may think it (HPV) infects the girls only because men don’t have a cervix*.*’*

*‘All teachers should be given the same information*. *For instance in our district*, *only the head-teacher and two other teachers were called… They can come and give a seminar to all the teachers and all the parents…’*

### Acceptability of HPV vaccine

Most of the respondents (89%, n = 302/339) would allow their daughter or close relative to receive the vaccine. Teachers in rural schools were more likely to accept the vaccine as compared to their counterparts in urban schools *(p =* 0.01).

Teachers who were aware of the initiative were more likely to accept the vaccine *(p =* 0.016). Similarly, those who accepted the vaccine had, on average, more knowledge about it than those who declined (mean score of 49.4% and 39.7% respectively, *p <*0.001).

There was no significant association between acceptability and the type of school (public or private), age, sex, level of education, length of service, religion, and marital status of the respondents.

**Fig 1:**
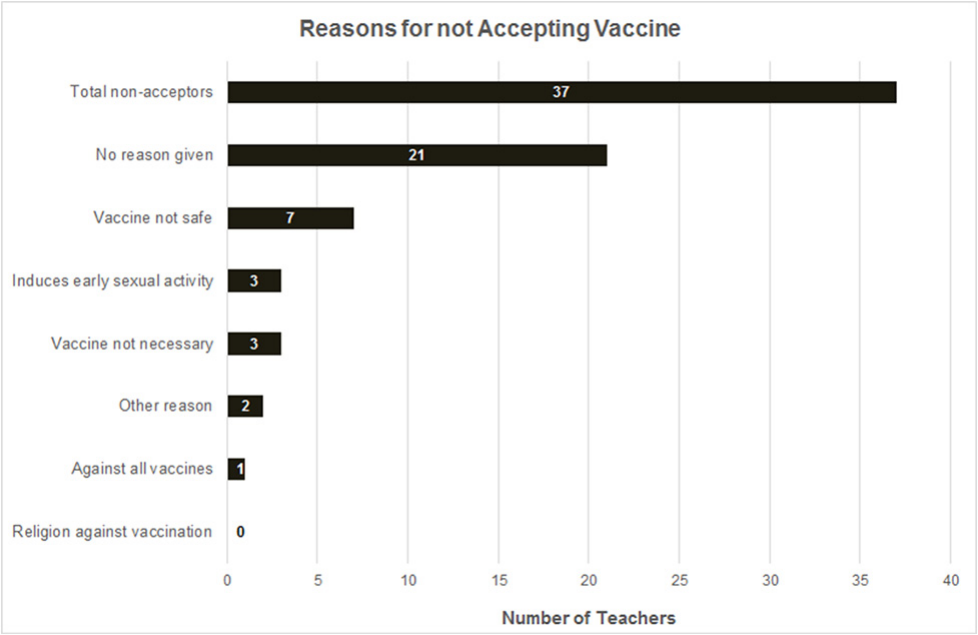
Reasons for not allowing daughter to receive HPV vaccination. Shows reasons why teachers would not allow a daughter to receive HPV vaccine.

About 11% (37/339) of the respondents reported that they would not allow their daughter or close relative to receive the vaccine. More than half did not give a reason for declining while seven of the 37 had concerns about vaccine safety. Fig 1 summarizes reasons for vaccine refusal.

All FGD participants would allow their daughter or close relative to receive the vaccine. They also reported that some parents and teachers had reservations or had rejected the vaccine. They cited lack of enough information and fear of side effects as the main concerns. Many of those who had reservations later accepted the vaccine once they got more information about it, as noted by an FGD participant quoted below:

*‘There are some (parents) who would come and ask us about it*, *we would explain to them why the vaccine is given*, *and then after explanation*, *they would accept that their children be given the vaccine*.*’*

The participants did not identify any particular side effects that they feared the vaccine might cause. Only one school reported actual side effects where two girls felt dizzy. This favorable safety profile reduced their fears for subsequent doses.

Another concern was fear among some parents and some teachers that the vaccine was a contraceptive. Most (12/13) FGD participants had heard about this concern but no one could identify its origin. They reported that it is a fear that cuts across most vaccinations and one that could be allayed by proper information from health officials.

### Respondents’ attitudes towards the HPV vaccine

**Fig 2:**
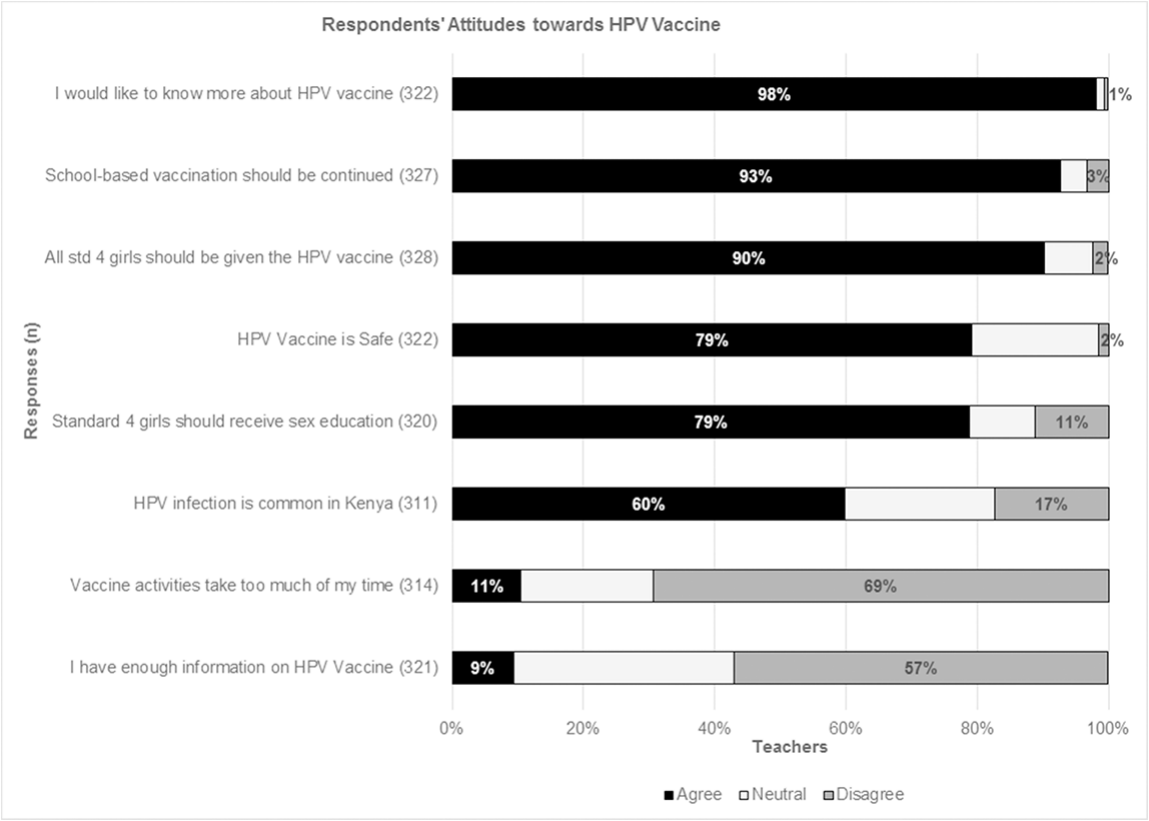
Participants’ responses to various statements. Shows participants’ responses on a Likert scale: The black color represents those who ‘agreed’, the light grey color represents the proportion who were ‘neutral’ and the dark grey represents those who ‘disagreed’ with the statement.

Respondents’ attitudes were measured on a scale of 1–3 (agree, neutral and disagree). Nearly all respondents (98%) expressed interest to know more about the HPV vaccine, 93% supported school-based vaccine delivery and 79% felt that the vaccine was safe (Fig 2).

### Barriers to successful implementation of the vaccination program:

Out of all respondents, 70% (237/339) cited at least one barrier that hindered the success of the vaccination project. A third of all respondents cited lack of information as a major barrier to HPV vaccination. Other prominent barriers were poor accessibility of the region (16%), pupil absenteeism (4%) and fear of side effects (8%).

**Fig 3:**
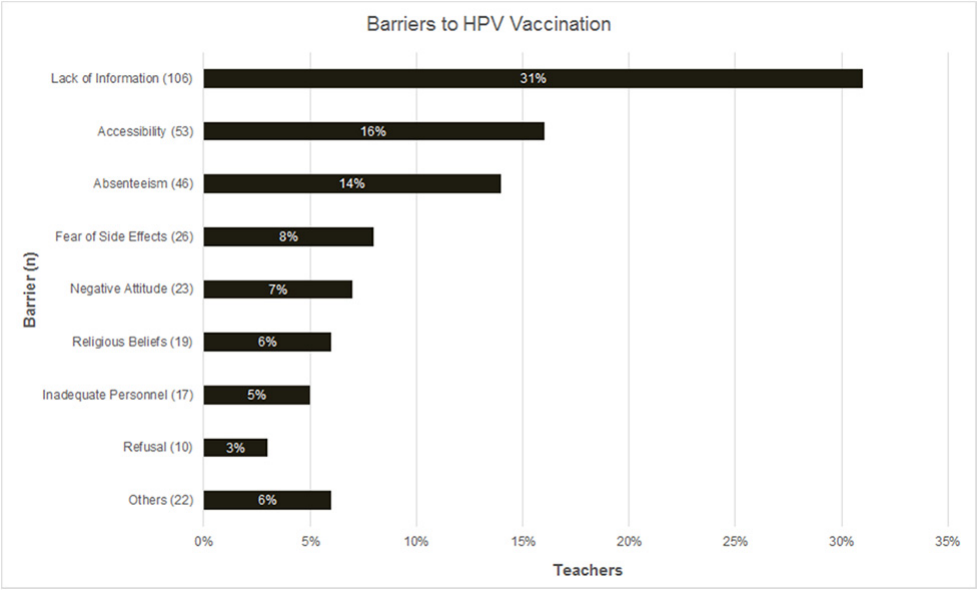
Barriers to the HPV vaccination project. Shows barriers to the HPV vaccination project in Kitui County as reported by Primary School teachers.

Less prominent barriers included negative attitude towards the vaccine by some parents or teachers, religious beliefs, refusal to be vaccinated by the girls or their parents, poor organization and planning, inadequate means of transport for MOH staff and dose delays past the expected vaccination dates (each cited by less than7% of the respondents) (Fig 3).

The participants reported that accessibility was a major issue due to poor road network and vastness of the County. They also observed that MOH vaccinators did not have adequate means of transport to traverse the County.

Some cited cultural and religious beliefs that were against vaccinations and fear of side effects as barriers too. One of the FGD participants made the following statement:

*‘We have some religions that don’t allow modern medicine*, *so the government should come in and decide what to do with the parent*.*’*

### Disruption of school activities

Most of the respondents (75%, n = 244/327) felt that the exercise only minimally disrupted the school activities. A fifth felt there was no disruption at all, while 6% thought the exercise caused severe disruption. Comparing teachers who thought there was at least some disruption (81%) and those that reported no disruption (19%), the latter were more likely to accept the vaccine *(p =* 0.014).

### Consenting to vaccinate

FGD participants felt that it was desirable for the parent and girl to give consent before vaccination. However, most (10/13) FGD participants felt that all girls should be vaccinated whether the parent/guardian agrees or not. Many felt that since the government had initiated the vaccine, it was beneficial to the girls and should be given by all means including enforcement by the police. Citing an example, a few mentioned a religion called ‘*Kavonokya’* whose followers do not take any form of modern medicine and are often forced to allow their children to receive the regular vaccines. One of the FGD participants made the following remark:

*‘…I had another case unrelated to HPV vaccine*. *In my former school a child from ‘Kavonokya’ religion was sick*. *The mother had died of anemia and the child was so sick he couldn’t concentrate [in class]; we had to force the child to go to hospital*. *So for the benefit of the child we have to force [vaccination]*.*’*

## Discussion

The aim of this study was to assess the knowledge and acceptability of HPV vaccine among primary school teachers in Kitui County Central Division and explore their views on facilitators and barriers of HPV vaccination following the launch of the first HPV pilot program in Kenya by the MOH. We found high levels of awareness (90%) and acceptability (89%) of HPV vaccine, and a moderate level of knowledge about the vaccine, HPV, and cervical cancer among the teachers (48%). Teachers, together with adolescents, parents and healthcare professionals, are key facilitators to successful vaccination programs [16,19,21,29,48]. Our findings on awareness of HPV vaccine concur with those from studies conducted after an awareness campaign in other parts of the world. Levels of 91% awareness among parents of adolescent daughters have been reported in North America [49] and France [50]. High levels of awareness of HPV vaccine among adolescent women, parents and nurses have also been reported in Cameroon following an education campaign [20,51,52] although most studies in SSA report generally low levels of awareness among key facilitators [21,53]. In a qualitative study in Tanzania, none of the parents, teachers and girls interviewed knew of the vaccine [19]. Similarly, in another study on women attending two hospitals in Kisumu, Kenya, none of the women had heard of the HPV vaccine [25]. The high level of awareness among teachers in our study was most likely due to the ongoing HPV vaccination campaign in the County. Teachers in rural schools were more likely to be aware of the vaccination initiative than their counterparts in urban schools. This is likely because rural schools had fewer teachers per school than urban schools making it easier to disseminate information on the vaccine by word of mouth.

Findings on acceptability are consistent with results from several studies in SSA and many other parts of the world showing high acceptability of the HPV vaccine or willingness to recommend it to a friend or relative. Previous studies in the United Arab Emirates, Argentina, Ghana, Cameroon, Tanzania, Uganda and Kenya reported high acceptability levels ranging from 75% to 99% [17,19,20,53–56].

A recent study in Nairobi reported that 69% of women knew that Pap smear testing is used to screen for cervical cancer [17], similar to 70% in our study. However, very few women in Kenya actually access screening services [17]. More than half of the teachers in this study (56%) do not know that screening by Pap smear is necessary even after one has received the HPV vaccine. Currently available bivalent and quadrivalent HPV vaccines which cover two oncogenic HPV serotypes (HPV 16 and 18) prevent about 70% of cervical cancer cases [57,58] and a nonavalent vaccine which covers five additional oncogenic HPV genotypes (HPV 31, 33, 45, 52 and 58) prevents up to 90% of all cervical cancer [59]. In addition, the vaccines are not effective if administered after infection has occurred. Therefore, vaccination against HPV does not obviate the need for cervical cancer screening.

The fact that female teachers in this study had more knowledge on HPV and cervical cancer than male teachers (50% versus 46%, *p =* 0.002) may be due to a perception that HPV affects women only. This is similar to findings of a study among secondary school teachers in Malaysia where awareness of HPV vaccine was higher in female teachers than in males (54% versus 33%) [48].

Noted barriers to HPV vaccination program include insufficient information, poor road access and pupil absenteeism. Kitui County has a poor road network with only 0.3% of roads paved [46], and is reflective of many other areas in Kenya. This creates challenges in accessing populations and transporting vaccines and health personnel in rural areas [60]. Pupil absenteeism on the vaccination days is also noted as a major barrier in a study in Tanzania [16]. This shows that good record keeping and tracking will be required to reach girls who may transfer or drop out of school before completing the three vaccine doses. Poor transport system and absenteeism calls for continued effort towards innovative delivery strategies and vaccine design to create heat-stable, single-dose HPV vaccines. Teachers’ concerns about side effects in our study are minimal (11%) compared to 35% of males in a study in Tanzania where head teachers in private schools would not allow vaccination out of fear of parents [19].

School-based delivery is the most preferred method for reaching girls with the HPV vaccine and has been used in Rwanda, South Africa, Tanzania and Uganda with high levels of uptake [21, 26]. Other studies in Botswana [61] and Ghana [46] recommended schools as the ideal venue. However few studies have assessed teachers’ perceptions about HPV vaccine. To the best of our knowledge, this is the only study done in Kenya that assesses teachers’ knowledge on HPV vaccine. Assessing and securing teachers’ support will be essential to successful vaccine implementation in Kenya.

Due to resource constraints, this study was conducted in one of 20 divisions in Kitui County. Although there are no discernible differences (in socio-economic levels, urbanicity, ethnicity or school distribution) in other divisions, collecting data from selected schools across the entire County may have provided a more complete picture on teachers’ views for national utility. Willingness to recommend the vaccine to a daughter or close relative was used as a marker for vaccine acceptability. This may not necessarily reflect the real picture on uptake of the vaccine at the time of administration. Another limitation was the fact that our questionnaire was not validated before use. In addition, the significant number (38%) of teachers who did not participate in the study may be a potential cause of bias. The main strength of this study was the use of qualitative methods to provide further insight into quantitative data.

## Conclusion

Our study shows that teachers have embraced the school-based approach as a mode of giving adolescent girls the HPV vaccine. Future campaigns should leverage this support by teachers. The study highlights the gap in knowledge on cervical cancer and HPV vaccines reported by similar studies. It also shows that insufficient knowledge on HPV vaccine may reduce the willingness of teachers to allow their daughters to be vaccinated or recommend the vaccine to others. There is need to address vaccine safety concerns and educate the community that HPV is a sexually transmitted infection that affects both men and women. As the country prepares to launch a nation-wide HPV vaccination for adolescent girls, one of the key investments should be in dissemination of information on HPV, HPV vaccine and cervical cancer. Effective ways of educating teachers, parents and girls are therefore needed. Because schools are likely to be the chosen vehicles/venues for delivery the first step should be recruitment and training of teachers to act as vaccine champions so as to educate their colleagues, parents and targeted girls. In addition, community mobilization strategies such as targeting audiences in social and religious gatherings as well as use of mass media may be viable strategies to disseminate information on HPV vaccine and cervical cancer to facilitate successful implementation.

## Supporting Information

S1 Dataset Dataset set from HPV vaccine study in Kitui, Kenya. (CSV)

S1 Text Questionnaire for HPV vaccine study in Kitui, Kenya. (PDF)

S2 Text Focus Group Guide for HPV vaccine study in Kitui, Kenya. (PDF)
