# Supplementary material for: Knowledge on HPV Vaccine and Cervical Cancer Facilitates Vaccine Acceptability among School Teachers in Kitui County, Kenya
Source: PLoS One. 2015 Aug 12;10(8):e0135563. doi: 10.1371/journal.pone.0135563 (PMC4534439; doi:10.1371/journal.pone.0135563)
Supplement: S1 Text — (PDF) [file pone.0135563.s002.pdf]

QUESTIONNAIRE

STUDY TITLE:A teachers' perspective on School-based HPV Vaccination in Kitui County: Knowledge, Acceptability, Barriers and Opportunities

SCHOOL DETAILS:

1. Name of your school:____

2. Type of Institution: ☐ Public ☐ Private

3. Location of School: ☐ Rural ☐ Urban

DEMOGRAPHICS:
Please fill in the following information about yourself:

4. What is your age? ____________ Years.Date of Birth:____________ (Year)

5. Sex: ☐ Male ☐ Female

6. Position:

   ☐ Head-teacher ☐ Deputy Head-teacher ☐ Teacher

   ☐ Other (specify):___________________

7. Level of education attained so far:

   ☐ KCSE ☐ Certificate

   ☐ Diploma ☐ Degree

8. Years of Service:

   ☐ 0- 5yrs ☐ 5-10 yrs ☐ 10-20 yrs ☐ More than 20 yrs

9. What is your religion?

   ☐ Catholic ☐ Muslim

   ☐ Protestant. Which denomination? ____________________________

   ☐ Other: ____

10. What is your marital status?

   ☐ Single ☐ Married ☐ Divorced ☐ Other (Specify):______________

11. What is the estimated average age of girls in standard four in your school?

   ☐ 8 years or younger ☐ 9-11 years

   ☐ 12-14 years ☐ 15 years or older

12. Are you aware that all standard four girls in Kitui County are being offered a Human Papillomavirus **(**HPV) vaccine? ☐ Yes ☐ No

If YES, how did you hear about it? (Tick all that apply)

☐ from Fellow teachers ☐ On Radio

☐ from Education Officials ☐ On Television

☐ from Health Officials

☐ Other (specify): ______________________________

13. Who lead the HPV vaccine awareness campaign in your area?

☐ Ministry of Health Officials

☐ Ministry of Education Officials

☐ County Government Officials

☐ Don't Know

☐ Other:______________________________________

14. Who would you prefer to lead such an awareness campaign in the future?

☐ Ministry of Health Officials

☐ Ministry of Education Officials

☐ County Government Officials

☐ I don't know

☐ Other (specify):____________________________________

15. Did your school participate in the HPV vaccine awareness campaign?

☐ YES ☐ NO ☐ I don't know

If YES, who was involved in the awareness activities? *(Tick any/all that apply)*

☐ Pupils

☐ Parents

☐ Teachers

☐ Head-teacher/ Deputy Head-teacher

☐ Other (specify):____________________________________

16. (a) Did your school get any promotional materials (brochures, posters, pamphlets, etc) on HPV?

☐ YES ☐ NO ☐ I don't know

(b) If YES, what did the material contain? *(Tick any/all that apply)*

☐ Information on transmission of HPV

☐ Information on diseases caused by HPV

☐ Information on prevention of HPV

☐ Information on HPV vaccine

☐ Information on Cervical Cancer

☐ Don't Know

☐ Other (specify): ______________________________________

(c) Do you feel the content in these materials was sufficient?

☐ Sufficient ☐ Insufficient ☐ Don't Know

☐ Other (specify)______________________________________

17. Did you attend any seminar or training on HPV Vaccine?

☐ YES ☐ NO ☐ Other (specify): _________________________

18. What diseases does HPV Vaccine protect against (tick any/all that apply).

☐ Cervical Cancer

☐ Anal Cancer ☐ Other (specify):____________________

☐ Vulvar Cancer

☐ Warts

☐ HIV/AIDS

☐ Breast cancer

19. What is the mode of transmission of HPV? *(Tick any/all that apply)*

- ☐ Physical contact
- ☐ Aerosol/Air droplet
- ☐ Sexual intercourse
- ☐ Other (specify): ________________________________

20. Which of the following persons can be infected by HPV?

☐ Male ☐ Female ☐ Both ☐ I don't know

21. Nearly everyone infected with HPV will have symptoms:

☐ True ☐ False ☐ Don’t Know

22. Infection with HPV may lead to cervical cancer:

☐ True ☐ False ☐ Don’t Know

23. Cervical cancer is a leading cause of cancer deaths in women in Kenya:

☐ True ☐ False ☐ I Don’t Know

24. Have you heard about Pap smear test?

☐ Yes ☐ No ☐ Other (Specify):____________________

25. What is a Pap Smear test used for?

- ☐ Testing sexually transmitted diseases (STDs)
- ☐ Treating Cervical Cancer
- ☐ Cervical cancer Screening

26. There is no need for Pap smear screening after receiving HPV vaccination

☐ True ☐ False ☐ Don’t know

27. Would you allow your daughter or a close relative to get HPV Vaccination?

☐ YES ☐ NO ☐ I am not sure

If your answer is NO, please indicate why?

- ☐ I am against all Vaccinations
- ☐ The Vaccine is not safe
- ☐ The vaccine will make young girls start sexual activity early
- ☐ My religion does not allow vaccination
- ☐ The HPV vaccine is not necessary
- ☐ Other (specify): ____

28. What was the effect of vaccine- related activities on daily school/educational activities?

☐ No disruption ☐ Minimal disruption ☐ A lot of disruption

☐ Other (Specify):________________________________

29. Which of these delivery system do you think is appropriate for vaccine delivery

☐ Schools

☐ Health facilities/clinics

☐ Community (village market, churches)

☐ Other (Specify): ____

For the following section, please indicate whether you agree or disagree with the statement:

	**Agree**	**Neutral**	**Disagree**
30. All standard 4 girls should get the HPV Vaccine			
31. HPV Vaccine is safe			
32. HPV infection is common in Kenya			
33. I have enough information about HPV vaccine to guide my pupils			
34. I would like to know more about the HPV vaccine			
35. Standard 4 girls should get education about sex			
36. Vaccine-related activities eat too much of my teaching time			
37. School-based vaccination of children should be continued			

38. In your own view do you think HPV vaccination to standard four girls was successful in Kitui?

____

39. What in your opinion hindered the vaccination exercise in primary schools in Kitui?

40. What do you think can be done to improve the vaccination exercise in primary schools?

END OF QUESTIONNAIRE . **Thank you for your time**
