# Supplementary material for: Knowledge on HPV Vaccine and Cervical Cancer Facilitates Vaccine Acceptability among School Teachers in Kitui County, Kenya
Source: PLoS One. 2015 Aug 12;10(8):e0135563. doi: 10.1371/journal.pone.0135563 (PMC4534439; doi:10.1371/journal.pone.0135563)
Supplement: S2 Text — (PDF) [file pone.0135563.s003.pdf]

FGD Guide

Focus Group Discussion

STUDY TITLE: A teachers' perspective on School-based HPV Vaccination in Kitui County: Knowledge, Acceptability, Barriers and Opportunities

Date: ____________________ Time:__________________________

Venue:____

Number of participants: Male: ___________ Female: ___________

Name of Note-taker:____

1. Introduction:

Thank you everyone for coming.

My name is **Moses Masika.** I am a Masters Student at the University of Nairobi Institute of Tropical and Infectious Diseases (UNITID) and I will be facilitating this focus discussion.

My colleague, _______________________________ , shall be doing the recording and note taking.

The Purpose of this discussion is to explore facilitators, barriers and opportunities presented by HPV Vaccination of Standard four girls in Kitui County.

This discussion shall be recorded using a voice recorder and noted on paper also to ensure we capture everything that will be discussed. We shall keep it confidential as much as possible and we will not use your names in our reports. Our reports will only have quotes from the discussion. This discussion will take approximately 90 minutes. Your participation is voluntary and you are free to leave the discussion at anytime if you feel the need to do so.

Consent:

If you agree to participate, please sign the consent forms we have issued to you.

2. Ground Rules:

Before we start our discussion, I’d like us to agree on our ground rules:

(Group to suggest any ground rules they would like to have)

The following should be included:

1. Everyone should participate, there are no right or wrong answers, we are here to hear your opinions and get ideas from you.
2. Bring out all sides of any issue, both positive and negative
3. Confidentiality: ‘Everything we discuss here should remain here’
4. One person talks at a time—avoid interruptions
5. Phones on silent mode and avoid attending to them until after the discussion

Ice breaker: What are the vaccines that you are aware of that are given to young girls?

1. **Awareness:**

(a) What do you know about the ongoing government initiative to offer HPV vaccine to all class 4 girls in Kitui county?

(b) What is the level awareness about the HPV vaccine in schools?

(c) What are the main sources of information on HPV Vaccine?

(d) Which information sources do you prefer?

2. Acceptability & Uptake

(a). Has the HPV Vaccine been accepted by teachers? What about parents and students?

(b). How is the uptake among the girls in school?

(c). What are your views on drop outs (i.e. did not complete the vaccine?)

(d). What are the reasons for declining or dropping out?

3. Knowledge on HPV vaccine and cervical

(a). What do you know about HPV? Probe mode of transmission

(b). What are the consequences of HPV infection?
Probe for symptoms, warts, cervical cancer, other cancers?

(c). How can cervical cancer be prevented?

4. What is the average age of girls in standard four in this area?
Probe: Feeling on age-appropriateness for the vaccine.
Should younger or older girls in other grades be targeted?

5. Concerns and fears

What were you worried about concerning the HPV vaccine?
Probe: Do you have any safety concerns?
Did you witness any side-effects in the girls who were vaccinated?
What do you think about the age?
(Are standard four girls too young or too old to vaccinate)

6. What was the effect of vaccine-related activities on school activities?

Probe: Level of disruption

How was school activity disrupted

How can disruption (if any) be reduced?

7. Success

(a). How would you gauge the success of the vaccination project?

(b). What are the factors that facilitated the success of the vaccination process?

(c). What are the factors that hindered the success of the vaccination process?

8. Opportunities

(a). What do you think about offering other health promotion services during the vaccination campaign?

Probes: Would this be a platform to offer health education?

Would this be an appropriate platform to offer sex education?

Deworming?

Any other health services?

9. Improvement

(a). Which areas would you recommend the programmers to improve?

Probe: How/ in what way?

10. Parental Consent:

What action should be taken if parents decline to vaccinate their daughter?

Probe: Nothing?

Vaccinate her anyway?

Summary and closing remarks.

Thank you very much for your time.
